# Elderly patients with hormone receptor-positive HER2-negative metastatic breast cancer treated with CDK4/6 inhibitors in a multicentre cohort

**DOI:** 10.1007/s12094-024-03399-3

**Published:** 2024-03-22

**Authors:** Helena Pla, Eudald Felip, Verónica Obadia, Sonia Pernas, Gemma Viñas, Mireia Margelí, Roser Fort-Culillas, Sonia Del Barco, Nuria Sabaté, Eduard Fort, Clara Lezcano, Beatriz Cirauqui, Vanesa Quiroga, Agostina Stradella, Miguel Gil Gil, Anna Esteve, Sabela Recalde

**Affiliations:** 1 Department of Medical Oncology-Breast Cancer Unit, Institut Català d’Oncologia (ICO)-H.U.Doctor Josep Trueta, Girona, Spain; 2 Precision Oncology Group (OncoGIR-Pro), Institut d’Investigació Biomèdica de Girona (IDIBGI), Universitat de Girona, Girona, Spain; 3 Department of Medical Oncology-Breast Cancer Unit, Institut Català d’Oncologia (ICO)-H. U. Germans Trias i Pujol (HUGTiP), Badalona Applied Research Group in Oncology (B-ARGO), Badalona, Spain; 4 Department of Medical Oncology-Breast Cancer Unit, Institut Català d’Oncologia (ICO)-H.U.Bellvitge. Institut d’Investigació Biomèdica de Bellvitge (IDIBELL), Universitat de Barcelona, Barcelona, Spain; 5 Department of Pharmacy, Institut Català d’Oncologia (ICO)-H.U.Doctor Josep Trueta, Girona, Spain; 6 Department of Pharmacy, Institut Català d’Oncologia (ICO)-H.U.Bellvitge, Barcelona, Spain; 7 Department of Pharmacy, Institut Català d’Oncologia (ICO)-H. U. Germans Trias i Pujol (HUGTiP), Badalona Applied Research Group in Oncology (B-ARGO), Badalona, Spain; 8 Unitat d’Epidemiologia i Registre de càncer de Girona (UERCG), Institut d’Investigació Institut d’InvestigacióBiomèdica de Girona (IDIBGI), Universitat de Girona, Girona, Spain

**Keywords:** Elderly population, Real world, Metastatic breast cancer, CDK4/6 inhibitors, Toxicity

## Abstract

**Introduction:**

Cyclin-dependent kinases 4/6 inhibitors (CDK 4/6i) combined with endocrine therapy have become the gold standard in hormone receptor-positive (HR +) HER2-negative (HER2-) metastatic breast cancer (MBC). However, there is a significant lack of data regarding the efficacy and safety of these treatments in elderly patients. We present the results of a real-world data (RWD) cohort stratified by age at treatment initiation (≥ 70 years compared to patients < 70 years).

**Methods:**

Clinico-pathological data of HR + HER2- MBC patients who were candidates for CDK4/6i therapy between January 2017 and December 2020 at the Institut Català d'Oncologia (Spain) were retrospectively collected. The primary goal was to assess Progression-Free Survival (PFS), Overall Survival (OS), and safety outcomes within this patient population.

**Results:**

A total of 274 patients with MBC who received CDK4/6i treatment were included in the study. Among them, 84 patients (30.8%) were aged ≥ 70 years, with a mean age of 75, while 190 patients (69.2%) were under the age of 70, with a mean age of 55.7 years. The most frequently observed grade 3–4 toxicity was neutropenia, with similar rates in both the < 70 group (43.9%) and the ≥ 70 group (47.9%) (*p* = 0.728). The median Progression-Free Survival (mPFS) for the first-line CDK4/6i treatment was 22 months (95% CI, 15.4–39.8) in the < 70 group and 20.8 months (95% CI 11.2–NR) in the ≥ 70 group (*p* = 0.67). Similarly, the median PFS for the second-line CDK4/6i treatment was 10.4 months (95% CI, 7.4–15.1) and 7.1 months (95% CI 4.4–21.3) (*p* = 0.79), respectively. Median overall survival (mOS) was not reached either for the first- and second-line treatment.

**Conclusions:**

Our RWD suggests that elderly patients, when compared to those under 70, experience similar survival outcomes and exhibit comparable tolerance for CDK4/6i therapy.

## Introduction

CDK4/6i (palbociclib, ribociclib, and abemaciclib) in combination with endocrine therapy are the standard of care for HR + /HER2- MBC patients, excluding those with visceral crisis. Numerous studies have demonstrated the efficacy of these CDK4/6 inhibitors in terms of Progression-Free Survival (PFS) and Overall Survival (OS) [1–7.

The prevalence of breast cancer in elderly patients is increasing due to the increasing longevity of the population 8. Consequently, more than one-third of individuals diagnosed with invasive breast cancer, and nearly half of all breast cancer-related fatalities in Western societies, occur in individuals aged 70 and older 9. Moreover, elderly patients (≥ 70 years-) are frequently encountered in routine practice but are underrepresented in clinical trials 10. Real-world data (RWD) could provide evidence about the efficacy and safety of CDK4/6i in the elderly population, scarcely reported by randomized clinical trials (RCTs) [11–14.

Subgroup analyses of palbociclib, ribociclib, and abemaciclib in the elderly population have been previously reported [15–17. Existing data on efficacy suggest no substantial age-related dependency. Nevertheless, patients aged 75 and older exhibited elevated toxicity rates and necessitated more frequent dose adjustments due to comorbidities, frailty, and polypharmacy 18.

Regarding palbociclib, a subgroup analysis of the PALOMA-2 demonstrated that the PFS benefit was sustained in patients  ≥ 65 years old. Nevertheless, there is a lack of age-specific toxicity data in the first-line setting. Additionally, in the PALOMA-3 trial, toxicity was increased in the elderly population, although it did not reach statistical significance. A pooled analysis of PALOMA trials, which included only 10% of patients older than 75, did reveal increased myelosuppression in this subgroup; however, no safety concerns were identified, and no detrimental impact on PFS was observed 16.

Evidence regarding the utilization of ribociclib in elderly patients is relatively sparse. A published study compared the effectiveness and safety of first-line ribociclib in combination with letrozole, using a predefined age threshold of 65 years 17. Notably, anemia and fatigue were more prevalent among patients aged over 65, without any apparent compromise in treatment efficacy.

Finally, evidence regarding elderly patients treated with abemaciclib is also limited. Goetz et al. published results based on an age-specific subgroup analysis of the MONARCH 2 and 3 trials, reporting higher rates of adverse events in elderly patients but no differences in clinical outcomes 6.

This study aims to analyze the survival outcomes and toxicity in elderly patients (≥ 70 years old) treated with CDK4/6i in a real-world setting compared to patients < 70.

## Materials and methods

### Study population

This is an observational, retrospective cohort study conducted at the Institut Catala d’Oncologia (ICO), a specialized cancer center in Spain comprising ICO Hospitalet, ICO Badalona, and ICO Girona. The study included patients diagnosed with HR + /HER2- MBC patients who received treatment with CDK4/6i (both first and second line) between January 2017 and December 2020. Patients enrolled in clinical trials were excluded from the study. Ethical approval for the study was obtained from the institutional review boards at each participating hospital. Clinical data, which were anonymized, were retrieved from electronic medical records and oncological treatment prescription software.

We collected the following data: age at the initiation of treatment, Eastern Cooperative Oncology Group (ECOG) performance status, menopausal status, estrogen receptor (ER) and progesterone receptor (PR) expression, Ki67 value, type of endocrine partner (aromatase inhibitor or fulvestrant), specific CDK4/6 inhibitor used, treatment line (first or second), de novo or recurrent disease status, sites of metastasis, previous therapies, radiological treatment responses, at least one dose reduction, toxicity assessments, treatment initiation date, progression date, and date of death (when applicable).

Toxicity assessments were graded in accordance with the Common Terminology Criteria for Adverse Events (CTCAE) version 5.0, with a particular focus on hematological, hepatic, and pulmonary toxicities based on previously reported data.

The cohort study was stratified based on age at treatment initiation, with patients categorized as either ≥ 70 or < 70 years old. We selected the age of 70 as the cut-off point in accordance with the guidelines of the International Society of Geriatric Oncology for conducting geriatric assessments in elderly breast cancer patients. 19.

### Statistical analysis

Characteristics of the participants were described by means of counts and percentages for categorical variables, and medians and interquartile ranges (IQRs) for continuous ones, and were compared between groups of patients (≥ 70 years old vs.  < 70) using the Chi-square test and Mann–Whitney U test, respectively. Overall survival (OS) was calculated from the date of treatment initiation to the date of death, last follow-up, or administrative censoring (1st December 2021). Similarly, Progression-Free Survival (PFS) was calculated from the date of treatment initiation to the date of progression, death, last follow-up, or administrative censoring (1st December 2021), whichever occurred earlier. The Kaplan–Meier method was used to estimate median PFS and median OS with their 95% confidence intervals (CIs) and compared between strata using the log-rank test. All statistical analyses were conducted using R 4.1.1 software.

## Results

### Patient characteristics

A total of 274 patients diagnosed with HR + /HER2- MBC initiated CDK4/6i-based therapy between January 2017 and December 2020. Baseline patient and tumor characteristics are summarized in Table 1. The median follow-up period was 21.9 months (IQR 11.7–32.5), with a mean age of 62 years (IQR 52.0–71.5). Among these patients, 84 (30.8%) were aged ≥ 70 years, with an average age of 75 (IQR 72.0–78.0) within this subgroup, while 190 patients (69.2%) were younger than 70 years old, with a mean age of 55.7 (IQR 48.8–62.0). Notably, differences in performance status assessed by the ECOG scale were observed. The younger group had a higher percentage (47.3%) of patients with an ECOG status of 0 compared to the elderly group (23.6%). Furthermore, 26.6% of the entire study population received a diagnosis of de novo metastasis, with a consistent percentage across all age groups.

**Table 1:** Patient characteristics of a cohort of *n* = 274 metastatic breast cancer patients treated with CDK4/6 inhibitors

	Total *n* = *274*	< 70* n* = *190*	≥ 70* n* = *84*	*p*
Age, median [IQR]	62.0 [52.0;71.5]	55.7 [48.8;62.0]	75.0 [72.0;78.0]	< 0.001
ECOG, *n* (%):				< 0.001
0	97 (40.2%)	80 (47.3%)	17 (23.6%)	
1	123 (51.0%)	81 (47.9%)	42 (58.3%)	
2	21 (8.7%)	8 (4.7%)	13 (18.1%)	
Line of treatment, *n* (%				0.191
First line	208 (75.9%)	149 (78.4%)	59 (70.2%)	
Second line	66 (24.1%)	41 (21.6%)	25 (29.8%)	
CDK 4/6 inhibitor, *n* (%):				0.419
Palbociclib	203 (74.1%)	137 (72.1%)	66 (78.6%)	
Ribociclib	52 (19.0%)	40 (21.1%)	12 (14.3%)	
Abemaciclib	19 (6.9%)	13 (6.8%)	6 (7.1%)	
Hormone therapy, *n* (%):				0.011
Aromatase inhibitor	181 (66.3%)	135 (71.4%)	46 (54.8%)	
Fulvestrant	92 (33.7%)	54 (28.6%)	38 (45.2%)	
M1 de Novo, *n* (%)	73 (26.6%)	48 (25.3%)	25 (29.8%)	0.530
Estrogen receptor, *n* (%):				0.065
< 1–80%	136 (50.0%)	102 (54.0%)	34 (41.0%)	
≥ 80%	136 (50.0%)	87 (46.0%)	49 (59.0%)	
Progesterone receptor, *n* (%)				0.065
< 1–80%	104 (38.8%)	70 (37.4%)	34 (41.0%)	
≥ 80%	164 (61.2%)	117 (62.6%)	47 (58.0%)	
Ki-67, *n* (%):				0.976
< 10	19 (8.8%)	13 (8.7%)	6 (9.1%)	
10–20	67 (31.0%)	46 (30.7%)	21 (31.8%)	
> 20	130 (60.2%)	91 (60.7%)	39 (59.1%)	
Metastasis location, *n* (%)				
Hepatic, *n* (%)	66 (24.1%)	43 (22.6%)	23 (27.4%)	0.487
Lung, *n* (%)	68 (24.8%)	46 (24.2%)	22 (26.2%)	0.843
Bone, *n* (%)	204 (74.5%)	151 (79.5%)	53 (63.1%)	0.007

Patients were stratified into two groups at age of treatment initiation: < 70 (*n* = 190) and ≥ 70 (*n* = 84)

Among the total cohort, 208 patients (75.9%) received CDK4/6 inhibitors (CDK4/6i) as first-line treatment, while 66 patients (24.1%) initiated CDK4/6i as second-line therapy. Palbociclib was administered to 74.1% of patients, ribociclib to 19.0%, and abemaciclib to 6.9%, with no discernible clinical disparities observed among the treatment groups. Significant variations in the utilization of hormone therapy were noted between the two age groups, with fulvestrant being more commonly prescribed in the elderly cohort (45.2% vs. 28.6%, *p* = 0.011). However, there were no statistically significant differences observed in the percentage of the ER, PR, or Ki-67 expression and the presence of de novo metastatic disease.

Regarding metastasis site involvement, a higher incidence of bone metastasis was observed in the < 70 age group (79.5% vs. 63.1%, *p* = 0.007). However, no differences were found in hepatic or lung involvement (Table 1).

### Efficacy data

At time of analysis, 47.8% patients remained progression-free, being a stable disease the most frequent radiological response (48.5% of those  < 70, and 36.1% of those  ≥ 70). There were no significant differences in the rates of disease progression nor in overall responses between both age groups. Treatment with CDK 4/6i achieved an objective response rate (partial response and complete response) of 31.8%, with no differences between both age groups (30.5% vs. 34.8%). Clinical benefit (includes response and stable disease maintained  ≥ 24 weeks) was 76.6% in the whole cohort, with no statistically significant differences between the two populations, 79% in  < 70 years old and 70.9% in ≥ 70 years (Table 2).

**Table 2:** Outcomes in a cohort of *n* = 274 metastatic breast cancer patients treated with CDK4/6 inhibitors

	Total *n* = *274*	< 70* n* = *190*	≥ 70* n* = *84*	*p*
Progression, *n* (%):				1.000
No progression	131 (47.8%)	91 (47.9%)	40 (47.6%)	
Progression	143 (52.2%)	99 (52.1%)	44 (52.4%)	
Best response, *n* (%):				0.270
Progression disease	53 (22.2%)	34 (20.4%)	19 (26.4%)	
Stable disease	107 (44.8%)	81 (48.5%)	26 (36.1%)	
Partial response	66 (27.6%)	44 (26.3%)	22 (30.6%)	
Complete response	10 (4.2%)	7 (4.2%)	3 (4.2%)	
Not available	3 (1.3%)	1 (0.6%)	2 (2.8%)	

Patients were stratified into two groups at age of treatment initiation: < 70 (*n* = 190) and ≥ 70 (*n* = 84)

**Fig. 1:**
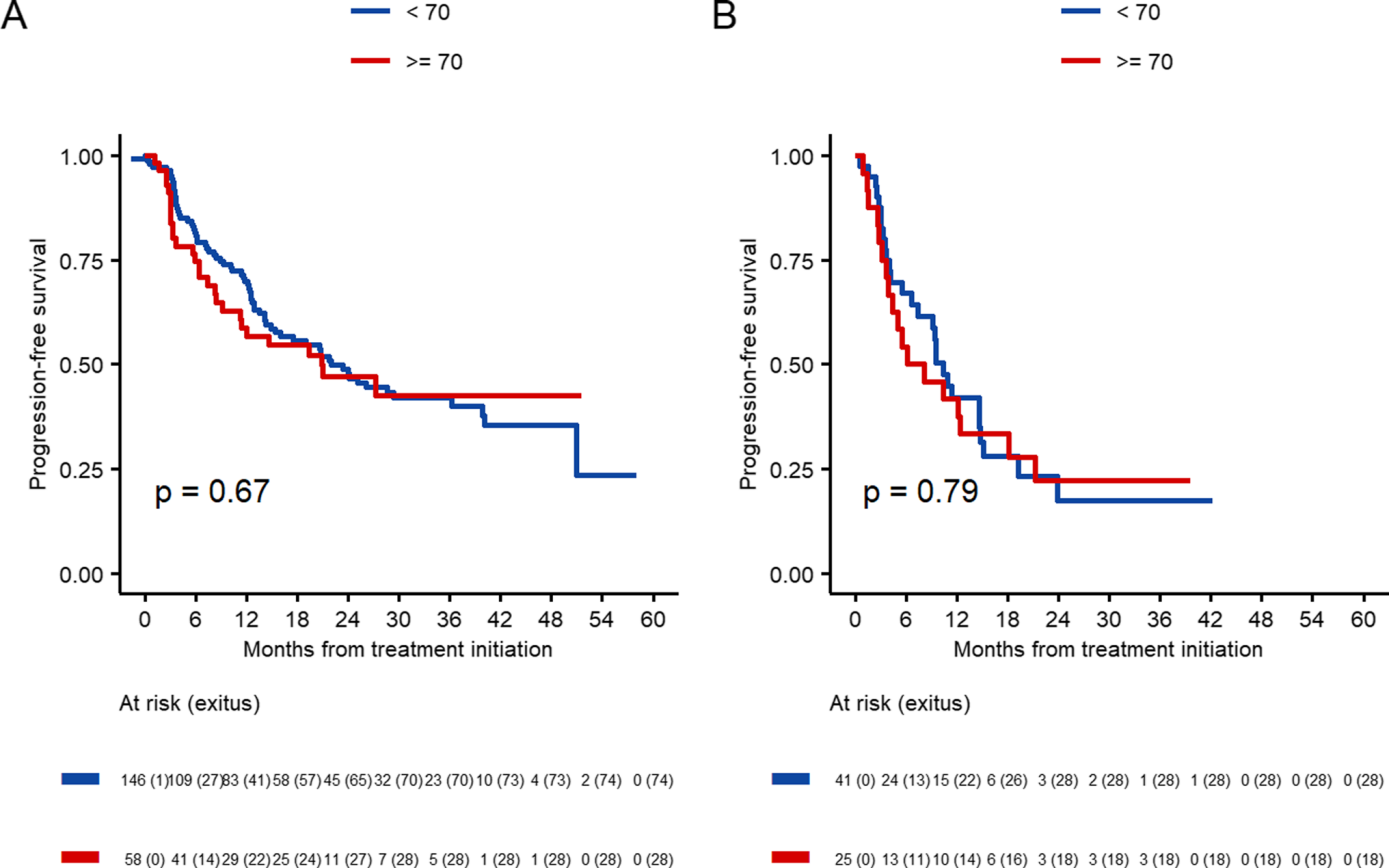
Progression-free survival according to age groups (< 70 years old or ≥ 70 years old). **A** Patients receiving CDK4/6i in first line. **B** Patients receiving CDK4/6i in second line

The PFS on the first line of treatment was 22 months (95% CI 15.4–39.8) in the  < 70 years old group, and 20.8 months (95% IC 11.2–NR) in the  ≥ 70 group (*p* = 0.67). A reduction in median PFS on the second-line treatment was observed, with 10.4 months (95% CI, 7.4–15.1) and 7.1 months (95% IC 4.4–21.3) Importantly, no statistically significant differences were detected between these groups (*p* = 0.79) (Fig. 1).

According to the ECOG scale, patients with ECOG 0 have a median in PFS of 23.4 months and 27.2 months in the younger and elderly group, respectively.

**Fig. 2:**
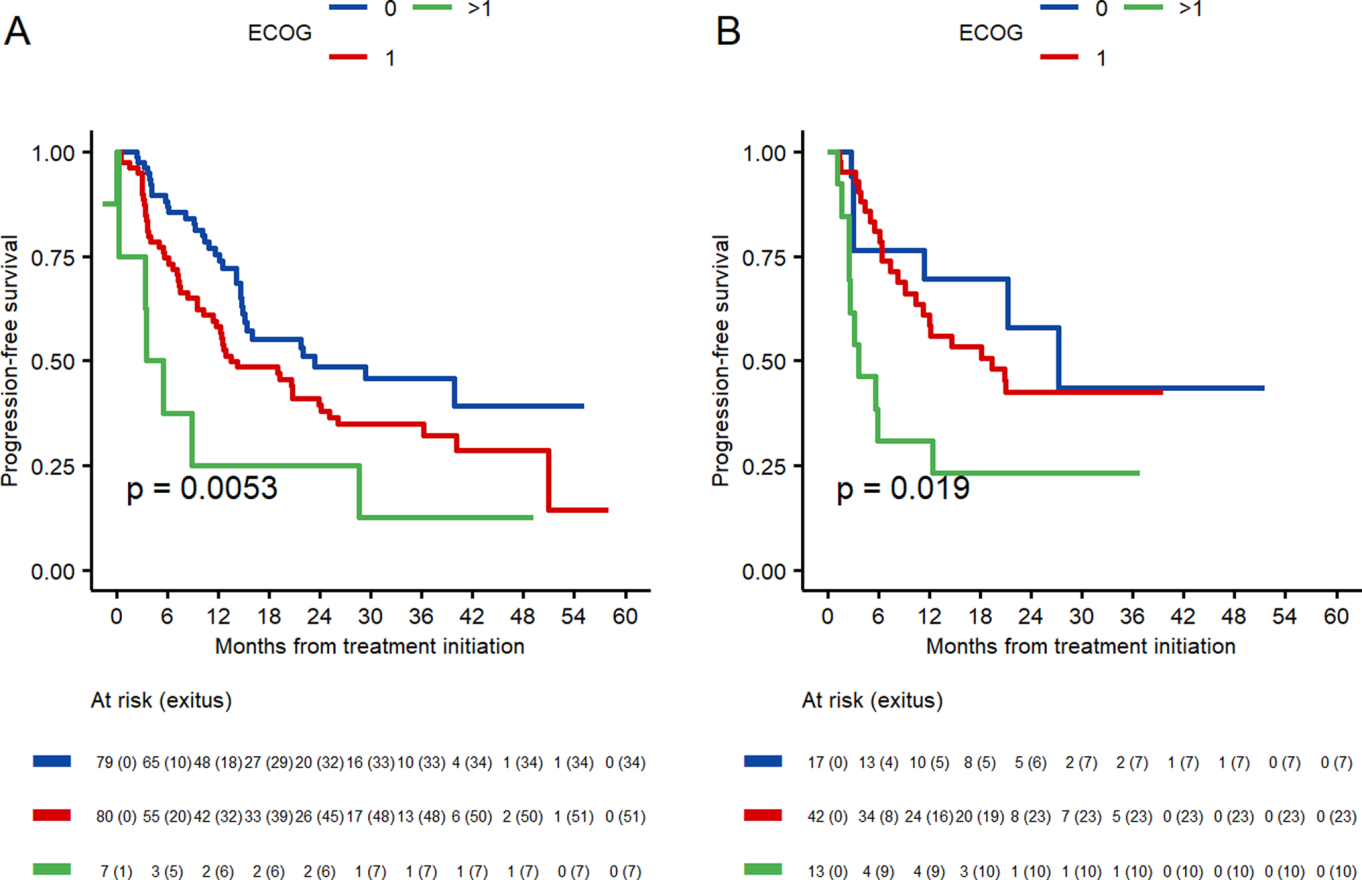
Progression-free survival according to ECOG status. **A** Patients < 70 years old. **B** Patients ≥ 70 years old

The ECOG performance status has an impact on both patients younger than 70 years old and those who are older. Patients with an ECOG score of 0 experience a longer Progression-free survival (PFS) in both age groups, with 23 months for the younger group and 27 months for the older group. Furthermore, patients with an ECOG score of 1 have a PFS of 13.5 months in the younger group and 19.4 months in the elderly group. Finally, patients with an ECOG score of 2 who are 70 or older have a PFS of 4.5 months, while those who are older than 70 have a PFS of 3.7 months. The differences according to the ECOG scale are statistically significant in both patient populations (*p* = 0.005 and *p* = 0.019, respectively) (Fig. 2).

**Fig. 3:**
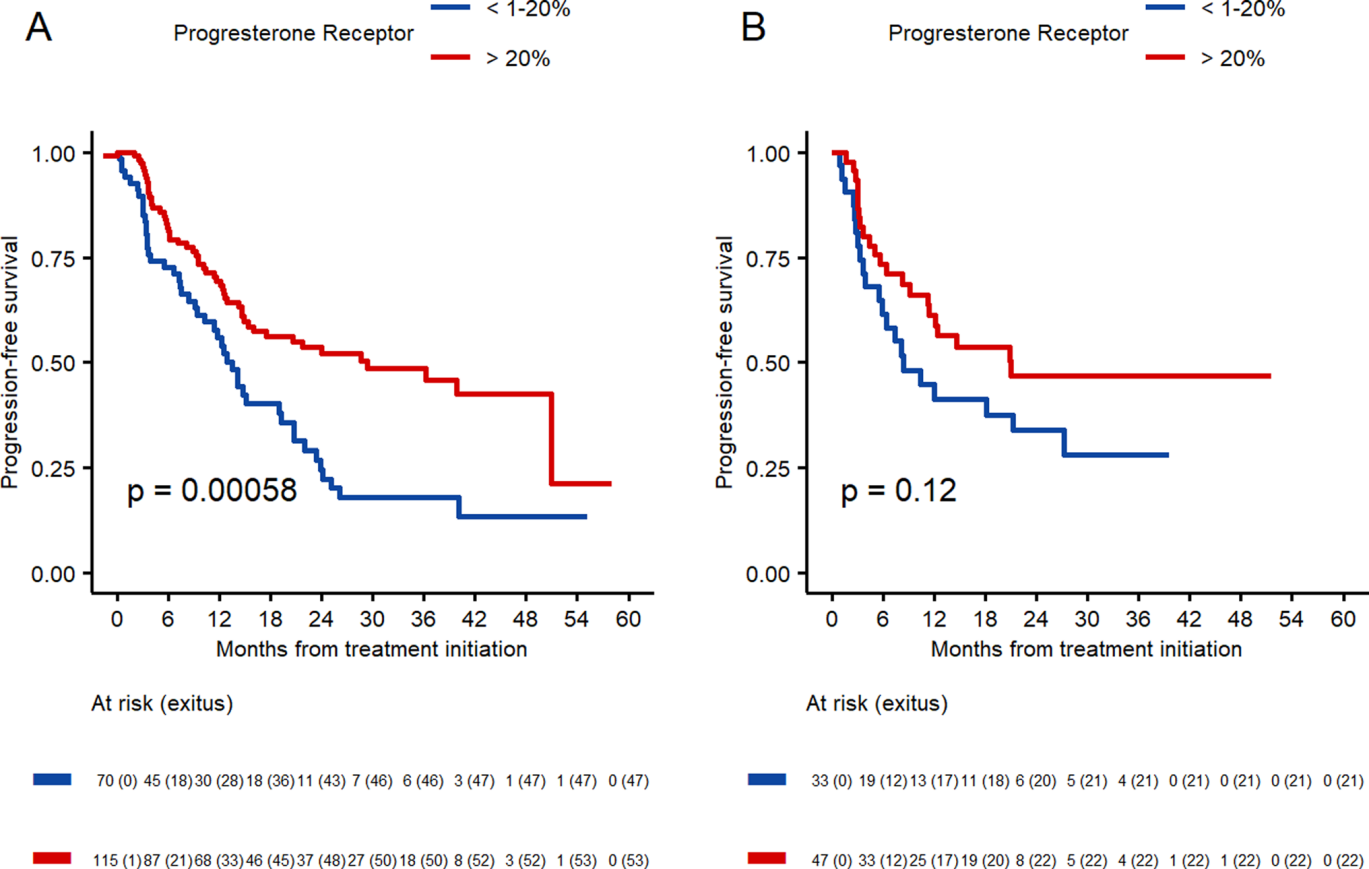
Progression-free survival according to the progesterone receptor expression. **A** Patients < 70 years old. **B** Patients ≥ 0 years old

**Fig. 4:**
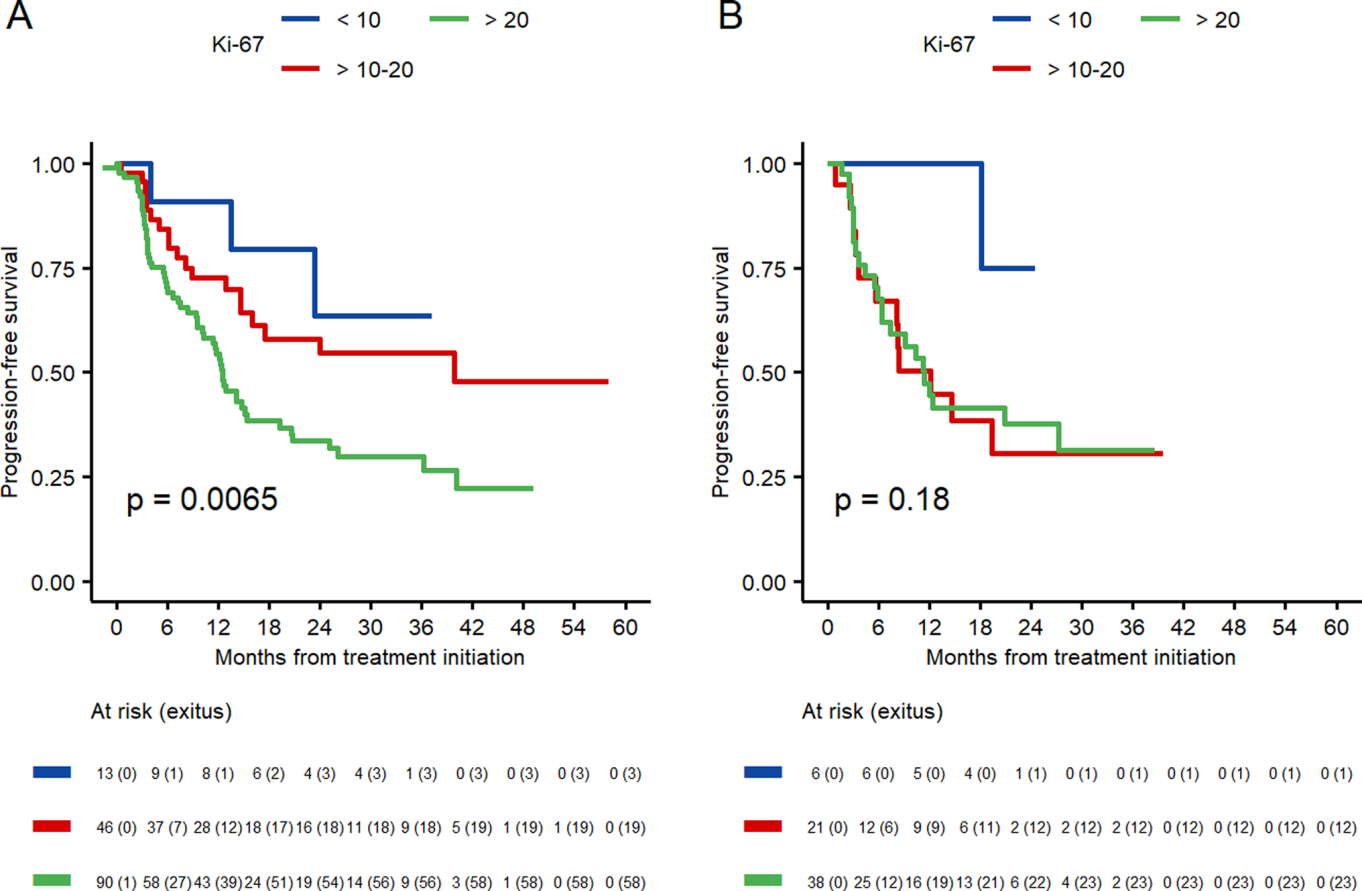
Progression-free survival according to Ki-67 expression by three groups, < 10, 10–20 and > 20). **A** Patients < 70 years old. **B** Patients ≥ 70 years old

Other clinical pathological factors impacting on PFS were the expression of PR and Ki-67. First, patients with PR < 20% experienced a shorter PFS both in the < 70 and the  ≥ 70 groups (13.5 m and 8.4 m, respectively), compared to the higher PR expression group (29.4 m and 21 m, respectively). Also, Ki-67 expression > 20% was associated with reduced PFS in both groups (12.5 m and 11.4 m). Patients with Ki-67 between 10 and 20% improved the PFS in both groups (39.8 m and 12.1 m), and finally, among patients with low-proliferation tumors (i.e., Ki-67 < 10%), longer PFS was observed, although without significant differences (Figs. 3 and 4).

Among 274 included patients, 124 (45.4%) experienced dose reductions, and these dose reductions did not have a negative impact on median PFS (mPFS). In fact, the mPFS of patients with dose reductions was significantly higher compared to patients without dose reductions in both groups: < 70 years old 25.1 months (95% CI 19–NR) vs. 14.1 months (95% CI 11.4–20.6), *p* = 0.005; ≥ 70 years old 27.2 (95% CI 14.6–NR) vs. 7.4 (3.9–21.3), *p* = 0.014.

Median overall survival (mOS) was not reached either for first- and second-line treatment. 91% of patients < 70 in first-line treatment were alive at 12 months, 81% at 24 months and 73% at 36 months, while for patients ≥ 70, the percentages were 83%, 77%, and 77%, respectively.

**Fig. 5:**
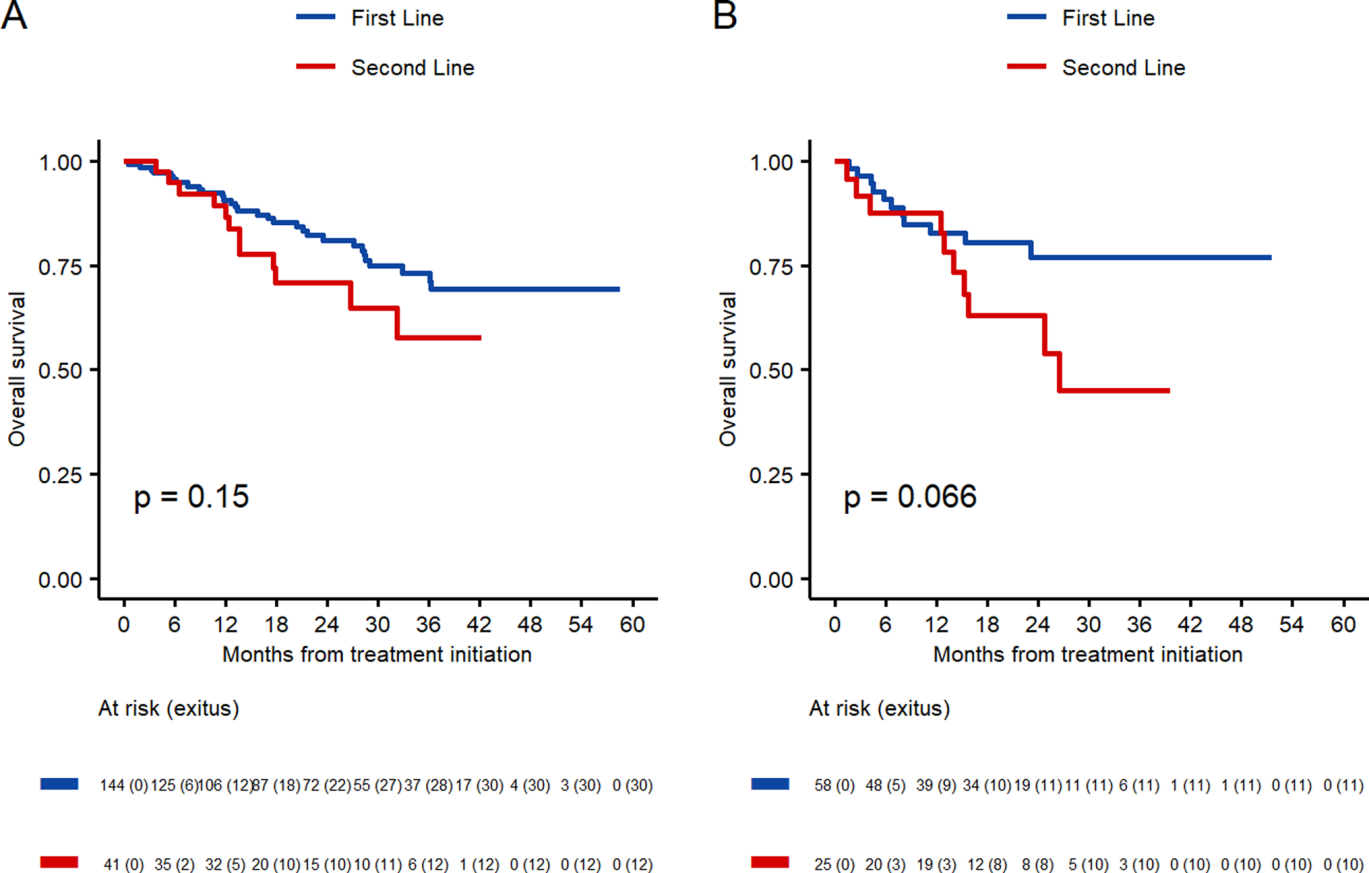
Overall survival by number of line receiving CDK4/6 inhibitor. **A** Patients < 70 years old. **B** Patients ≥ 70 years old

As for those in the second-line treatment, the cohort of patients < 70 showed that 87% of patients were alive at 12 months, 71% at 24 months, and 58% at 36 months. The results for patients ≥ 70 were 88%, 63%, and 45%, respectively (Fig. 5).

### Toxicity

The majority of patients in both groups initiated treatment with the standard dose of the CDK4/6i (97.8% of the total cohort, 98.4% of those < 70, and 96.4% of those ≥ 70), without statistically significant differences between the two groups (*p* = 0.375). Furthermore, regarding dose reduction due to side effects, 45.4% of the cohort needed a reduction during the treatment. Interestingly, age groups exhibited no significant differences (43.4% of those < 70, and 50.0% of those ≥ 70, *p* = 0.378). The proportion of patients who discontinued CDK4/6 inhibitors due to toxicity was 15.6% in the ≥ 70 year group and 11.6% in the < 70 year group (*p* = 0.373) (Table 3).

**Table 3:** Data on the initial dose and dose adjustments of CDK4/6 inhibitors among a cohort of *n* = 274 metastatic breast cancer patients

	Total *n* = 274	< 70 *n* = 190	≥ 70 *n* = 84	p
Initial dose, *n* (%):				0.375
Max dose	268 (97.8%)	187 (98.4%)	81 (96.4%)	
Adjusted dose	6 (2.2%)	3 (1.6%)	3 (3.6%)	
Dose reduction, *n* (%)	124 (45.4%)	82 (43.4%)	42 (50.0%)	0.378
Withdraw due to toxicity, *n* (%)	35 (12.8%)	22 (11.6%)	13 (15.6%)	0.373

Patients were stratified into two groups at age of treatment initiation: < 70 (*n* = 190) and ≥ 70 (*n* = 84)

There were no significant differences between the two age groups concerning hematologic toxicity. Neutropenia Grade 3–4 occurred in 45.1% of the total cohort, with similar rates in the < 70 group (43.9%) and the ≥ 70 group (47.9%) (*p* = 0.728). Liver toxicity also displayed no significant difference between both groups (*p* = 1.000), with grade 3–4 liver toxicity detected in 2.3% of the < 70 group. Finally, grade 3–4 lung toxicity was infrequent, occurring in 1.2% of the < 70 group. No patients aged ≥ 70 experienced grade 3–4 liver or lung toxicities (Table 4).

**Table 4:** Toxicities among a cohort of *n* = 244 metastatic breast cancer patients treated with CDK4/6 inhibitors, divided by age: < 70 (*n* = 171) and ≥ 70 (*n* = 73)

	Total *n* = 244	< 70 *n* = 171	≥ 70 *n* = 73	
Any hematologic toxicity, *n* (%)	165 (67.6%)	116 (67.8%)	49 (67.1%)	1.000
Grade of hematologic toxicity, *n* (%):				0.728
G1–G2	54 (22.1%)	39 (22.8%)	15 (20.5%)	
G3–G4	110 (45.1%)	75 (43.9%)	35 (47.9%)	
Any liver toxicity, *n* (%)	16 (6.6%)	14 (8.2%)	2 (2.8%)	0.160
Grade of liver toxicity, *n* (%)				1.000
G1–G2	12 (4.9%)	10 (5.8%)	2 (2.7%)	
G3–G4	4 (1.6%)	4 (2.3%)	0 (0.0%)	
Any lung toxicity, *n* (%)	2 (0.8%)	2 (1.2%)	0 (0.0%)	1.000

## Discussion

This real-world cohort of the elderly population provides clinical information in scenarios where there is a lack of data coming from randomized clinical trials. This study analyses the influence on tolerance and efficacy of different CDK4/6i comparing two groups of patients based on age with a cut-off point of 70 years irrespectively of their comorbidities.

It is essential to emphasize that the primary objective of treatment in metastatic disease, irrespective of age, is palliative, with the goal of controlling tumor progression while preserving the performance status and quality of life of the patient. Generally, it has been reported that elderly patients with metastatic disease experience poorer survival outcomes compared to younger ones 20. Furthermore, in HR + /HER2- metastatic breast cancer (MBC), the use of targeted therapies in combination with endocrine treatments, known for their favorable tolerability profile, may extend the time before chemotherapy is required.

CDK4/6i in combination with hormone therapy have already demonstrated their enhanced efficacy in patients over the age of 65 when compared to hormone therapy alone and should be the treatment of choice 21. The International Society of Geriatric Oncology (SIOG) has also analyzed the impact of CDK4/6 inhibitors in elderly populations. SIOG concluded that CDK inhibitors appear to be equally effective in older as in younger patients (cut-off of 65 y/o) with limited adverse events and toxicity in older patients. Existing data suggest that older patients experience comparable efficacy, with either similar or slightly increased toxicity from these agents compared to younger individuals. SIOG also remarked on the importance of potential drug interactions, understanding that elderly populations may be more poly-medicated. However, it is crucial to consider the treatment goal for patients with MBC. This approach not only aids in maintaining a higher quality of life but also highlights the moral imperative of comprehensively understanding the toxicity and efficacy of these therapies in the elderly population, a demographic often underrepresented in clinical trials.

The present study with a real-world cohort showed that clinical outcomes align with the published literature, highlighting that no differences in PFS and OS were observed between age-based groups 22. Among patients ≥ 70 years, the median PFS in the first line was 20.2 months vs. 22 months in < 70. Real-world studies of first-line CDK 4/6 inhibitors combined with endocrine therapies have demonstrated a median PFS ranging from 18.7 to 21.3 months 23, 24. The results of PFS in the second line are consistent with those published in the pivotal studies 25 and other real-world data studies 26. We could conclude that all patients benefit equally regardless of the age at which they receive the treatment, since we have not observed statistically significant differences between age groups.

Remarkably, patients with better performance status at the beginning had longer PFS, as ECOG has been proven a prognostic factor in MBC 27 and must be a valuable measure for treatment choices. Patients aged ≥ 70 years often exhibit a higher burden of comorbidities, resulting in reduced performance status and a larger proportion of patients with ECOG 1 or 2, up to 75% in our study. It has been reported that in clinical trials, only 20% of patients are over the age of 65, and the majority of these individuals have an excellent performance status with ECOG 0 around 70% 21. This discrepancy between the elderly population represented in clinical trials and elderly population treated in cancer centers creates a significant bias when assessing the efficacy and toxicity of standard treatments for MBC in the population aged over 70.

Our findings also suggested that low PR expression and higher Ki-67 displayed worse PFS outcomes. These features have been extensively proven as high-risk factors in early HR + /HER2- breast cancer [28–30. Also in advanced disease, some studies have shown a prognostic role of some of these factors (Ki67, PR status), but there is less or no evidence of their predictive role for response to CDK4/6i in metastatic disease 31, 32.

However, our study demonstrates that luminal B-like tumors also have a worse prognosis than luminal A-like tumors when CDK4/6i is added to hormonal treatment. These data are in accordance with the published data of the CDK4/6i efficacy analysis according to the intrinsic subtypes based on PAM-50 in the pivotal trials 33.

No significant differences were found in the overall response rate between those < 70 and ≥ 70, suggesting that age does not significantly affect the rate of disease progression or the best response rate in MBC patients treated with CDK4/6 inhibitors.

Our study confirmed that routine care patients tolerated the combination of CDK4/6 inhibitors and HT well, as observed in the pivotal clinical trials. The most common toxicities were neutropenia and liver function derangement, but a few patients had to discontinue treatment due to adverse events. Contrary to what is reported in the literature, toxicity data were similar in the elderly population compared to younger patients, even regarding hepatic or pulmonary toxicities 15. No grade 3 or higher pulmonary or hepatic toxicities were observed in the cohort of patients  ≥ 70.

No negative impact in either PFS or OS was seen when dose reductions occurred 28. In PALOMA-3 and PALOMA-2 trials, 34% and 36% of patients had a dose reduction 34, whereas in other real-world studies appears to be lower 23, 35–38. In our cohort, dose reductions most frequently occurred due to myelosuppression toxicity, which is the most limiting toxicity for palbociclib and 75% of the patients in our cohort received palbociclib. The apparent benefit in PFS observed in those patients who had at least one dose reduction is probably due to a bias, since those patients who progress early in less time on therapy are less likely to have a dose reduction.

The usual development of new anti-target molecules in early phase trials might not be adequate for choosing the best dose. These trials were based on the traditional concept of chemotherapy that maximum doses are most effective. However, this might not be appropriate for the development of anti-target drugs, and our findings support the question of whether the development of new anti-target drugs should be based on the maximum tolerable dose.

The strength of our study is the large number of patients and clinical data collected from patients treated in a real-world setting. Limitations are inherent to the descriptive and retrospective design of the analysis. It is worth mentioning that most patients were treated with palbociclib as it was the first CDK4/6i approved in our country. In contrast, our study included few patients treated with abemaciclib, which makes it difficult to interpret the data regarding this CDK4/6i. Moreover, information related to geriatric assessment is lacking, since not all patients had a geriatric evaluation before treatment initiation.

## Conclusions

This study supports the use of CDK4/6i in elderly patients. Patients  ≥ 70 had no increased risk in toxicity, and efficacy was similar to patients < 70 years old. Therefore, CDK4/6i as first- or second-line treatment should be offered to patients  ≥ 70 years with HR + MBC. Furthermore, in patients who required a dose reduction, no negative impact on efficacy was observed. The real-world setting provides further information to make better clinical decisions to improve the treatment of patients with HR + HER2- MBC.

## Data Availability

The data that support the findings of this study are available from the corresponding author upon reasonable request. Restrictions apply to the availability of these data, which were used under license for this study.
